# Endotoxin removal by polymyxin B: is it a question of dose or duration or both?

**DOI:** 10.1186/s13054-019-2584-5

**Published:** 2019-09-04

**Authors:** Patrick M. Honore, David De Bels, Leonel Barreto Gutierrez, Sebastien Redant, Andrea Gallerani, Willem Boer

**Affiliations:** 1 ICU Department, Centre Hospitalier Universitaire Brugmann, Place Van Gehuchtenplein, 4, Brussels, Belgium; 2 Department of Anesthesiology, Intensive Care Medicine, Emergency Medicine & Pain Medicine, Ziekenhuis Oost-Limburg, Genk, Belgium

Srisawat et al. described a randomized controlled trial (RCT) in patients with endotoxin activity assay (EAA) level ≥ 0.6 [1]. The polymyxin hemoperfusion (PMX-HP) group received a 2-h PMX-HP treatment plus standard treatment for two consecutive days. At baseline, monocyte human leukocyte antigen (mHLADR) expression, CD11b, neutrophil chemotaxis, and clinical variables were comparable between groups [1]. The increase in mHLA-DR expression between day 3 and baseline was higher in PMX-HP patients (*P* = 0.027). The inverse was true for the mean change in CD11b between day 3 and baseline (*P* = 0.002) [1]. However, at day 28, there was no difference in clinical variables including mortality, intensive care unit (ICU)-free days, renal recovery, and major adverse kidney events (MAKE) [1]. In PMX-HP patients, 23% had gram-positive (GP) bacteria, similar to the 20% GP bacteria in the Euphrates study [2], and 49% gram-negative (GN) bacteria [1]. Endotoxin is present in the outer membrane of GN bacteria, and endotoxemia is typically associated with GN infection [3]. However, the gastrointestinal tract is a reservoir of “dormant” endotoxin, and perturbations of permeability in particular when associated with splanchnic hypoperfusion may cause translocation and circulatory shedding of endotoxin, therefore accounting for endotoxin presence in GP infections [3]. Measuring EAA levels will identify patients benefiting most from PMX-HP therapy. The Euphrates study included septic shock patients with EAA levels ≥ 0.6. No difference in 28-day mortality was observed except in those with a multiple organ dysfunction syndrome score above 9 and EAA levels between 0.6 and 0.9 [4]. In vivo experiments demonstrated that EAA levels exceeding 0.9 correlate with an endotoxin burden of more than 50 μg/mL, far exceeding the adsorptive capacity of the PMX-HP cartridge dose in this trial [4]. As endotoxin is lipidic by nature, it associates with lipid-rich particles [5] leading to false negatives for EAA and underdetection of endotoxin mass when using the Limulus amebocyte lysate (LAL) bioassay [5]. An alternative assay discovered by Pais de Barros et al. based upon mass spectrometer quantitative assay detects not only the EAA but also the actual endotoxin mass, suitable for monitoring and tailoring duration and frequency of endotoxin removal therapies, either alone or in association with the LAL assay [5]. Contrary to the LAL assay, this new quantitative assay demonstrated endotoxin in the circulation for more than 48 h, instead of only a few [5]. A study comparing both assays is ongoing [5]. The new Tigris study will start soon also. This study led by Klein et al. [4] is a RCT only focusing on patients with EAA level between 0.6 and 0.9 and excluding patients with EAA level above 0.9. This RCT will confirm or infirm the findings of the post hoc analysis of the Euphrates study when excluding from the analysis patients with an EAA level > 0.9.

## Data Availability

Not applicable.

## Abbreviations

RCT: Randomized controlled study
EAA: Endotoxin activity assay
PMX-HP: PMX hemoperfusion
mHLADR: Monocyte human leukocyte antigen
ICU: Intensive care unit
MAKE: Major adverse kidney events
GP: Gram-positive
GN: Gram-negative
LAL: Limulus amebocyte lysate
